# Therapeutic Application of Virtual Reality in the Rehabilitation of Mild Cognitive Impairment: A Systematic Review and Meta-Analysis

**DOI:** 10.3390/vision6040068

**Published:** 2022-11-18

**Authors:** Hyunjoong Kim, Jihye Jung, Seungwon Lee

**Affiliations:** 1 Seogwangju Chung Yeon Rehabilitation Hospital, Gwangju 72070, Republic of Korea; 2 Department of Physical Therapy, Gwangju Health University, Gwangju 62287, Republic of Korea; 3 Institute of SMART Rehabilitation, Sahmyook University, Seoul 01795, Republic of Korea; 4 Department of Physical Therapy, Sahmyook University, Seoul 01795, Republic of Korea

**Keywords:** virtual reality, mild cognitive impairment, cognitive function, rehabilitation

## Abstract

This review aimed to quantify the effect of therapeutic application of virtual reality (VR) on cognitive function in individuals with mild cognitive impairment (MCI). We searched for randomized controlled trials involving VR in the interventions provided to individuals with MCI. After searching four international electronic databases, we analyzed six studies involving 279 individuals with MCI. RevMan 5.4 was used for quality assessment and quantitative analysis. Therapeutic application of VR in individuals with MCI resulted in a significant improvement in cognitive function (mean difference = −1.46; 95% confidence interval: −2.53 to −0.39; heterogeneity: χ^2^ = 970.56, df = 18, I^2^ = 98%; and overall effect: Z = 2.67, *p* = 0.008). However, there was no significant improvement in the subcategories such as global cognition, working memory, executive function, memory function, and attention. In conclusion, feedback stimulation through VR has a potential value in improving cognitive function in individuals with MCI. However, on the basis of the results of the subcategories, a personalized VR program is required for the individual subcategories of cognitive function.

## 1. Introduction

Mild cognitive impairment (MCI) may be a precursor to dementia [1], a stage in which cognitive symptoms are not fully understood [2]. The prevalence of MCI in adults aged above 60 years ranges from 6.7% to 25.2% and varies according to age and educational level [3,4]. Divided attention, learning new information, verbal fluency, and reaction time tend to decline with normal aging [5]. However the diagnostic criteria for MCI also include changes in cognition, abnormal cognitive function in one or more areas, concerns about normal daily activities, and absence of dementia [6,7].

Early detection of MCI and appropriate interventions are very important since they can slow the progression to dementia or improve the symptoms [8]. The recommended non-pharmacological interventions for MCI include combined interventions with exercise and cognitive training [9,10]. Furthermore, studies using virtual reality (VR) for the prevention and treatment of MCI have been performed until relatively recently [11]. Exercise combined with VR showed significant improvement not only in physical function but also in cognitive function in normal elderly individuals, and there was a tendency to prefer this combination to general exercise [12,13].

With the development of VR technology, many studies have been conducted on MCI, and numerous systematic reviews have been published [14,15,16,17,18,19,20,21]. Systematic reviews have suggested that semi-immersive VR was more effective than immersive VR, and it showed significant improvement in global cognitive function and short-term memory. However, there was no significant improvement in other variables. Moreover, the effect size was not large, even for the variables with significant improvement. Therefore, we believed it necessary to classify and analyze the cognitive function in more detail to clarify controversial results.

Thus, we performed qualitative and quantitative analyses of the effect of VR on cognitive function in randomized controlled trials (RCTs) using the therapeutic application of VR for MCI.

## 2. Materials and Methods

### 2.1. Study Design

In this systematic review and meta-analysis, we aimed to perform qualitative and quantitative analyses based on studies involving therapeutic application of VR in individuals with MCI. A systematic review was performed according to the Preferred Reporting Items for Systematic Reviews and Meta-Analyses (PRISMA) guidelines. The review protocol was registered in the International Prospective Register of Systematic Reviews (PROSPERO) (number: CRD42022360635).

### 2.2. Search Strategy and Selection of Studies

#### 2.2.1. Inclusion Criteria

1. Participants
  Participants were individuals with MCI alone.
2. Intervention
  Interventions included VR alone or combined interventions.
3. Comparisons
  Activities that did not involve an intervention or did not include VR were selected for comparisons.
4. Outcomes
  To perform a meta-analysis, a comparative analysis was performed when there were three or more identical variables in the studies.
5. Types of studies
  Among different study designs, only RCTs were selected.

#### 2.2.2. Exclusion Criteria

Studies not published in English or studies not reporting the appropriate data were excluded. In addition, studies published before 2013 were excluded from the synthesis of relatively recent studies.

#### 2.2.3. Strategy for Literature Search

We searched for studies published since 2013 wherein the study protocol was registered in PROSPERO until September 2022. The searched keywords were as follows: ‘mild cognitive impairment’ AND (‘virtual reality’ OR ‘rehabilitation’) AND (‘cognition’ OR ‘cognitive function’) AND ‘randomized controlled trial.’

The databases used for the search included the Cumulative Index to Nursing and Allied Health Literature (CINAHL), Excerpta Medica Database (Embase), Medical Literature Analysis and Retrieval System Online (MEDLINE), and Physiotherapy Evidence Database (PEDro).

#### 2.2.4. Study Selection and Data Extraction

Studies searched in the aforementioned electronic databases were exported to Microsoft Excel (Microsoft, Redmond, Washington, USA), and duplicate studies were excluded. According to the PRISMA guidelines, the full text of each study was checked after reviewing the title and abstract. Finally, studies were selected through consultation among researchers (H.K., J.J., and S.L.) with experience in meta-analyses.

#### 2.2.5. Quality Assessment

Quality assessment was performed using the risk of bias (RoB) tool provided by RevMan 5.4 (The Cochrane Collaboration, Oxford, England). RoB is a tool consisting of seven items: random sequence generation, allocation concealment, blinding of participants and personnel, blinding of outcome assessment, incomplete outcome data, selective reporting, and other biases. Each of the seven items was rated as high (−), low (+), or uncertain (?) by the researchers. If there was no agreement on the results, a consultation process was required.

### 2.3. Strategy for Data Synthesis

The included studies were synthesized and analyzed using RevMan 5.4. We performed a quantitative analysis using mean differences (MDs), considering RCTs with no homogeneity at baseline. For studies wherein the standard deviation was not reported in the values describing change from baseline, correlation coefficients were extracted and calculated from the results of the studies using the same variables. Therefore, data on outcome measures were extracted as MDs and presented as a random effects model considering the heterogeneity. In addition, the chi-squared and I^2^ tests provided in the software were used for heterogeneity.

An I^2^ value greater than 75% was considered to indicate high heterogeneity, and a value below 40% was considered to indicate low heterogeneity [22]. Publication bias in the studies was displayed using funnel plots [23].

## 3. Results

### 3.1. Literature Search and Characteristics of the Included Trials

**Figure 1:**
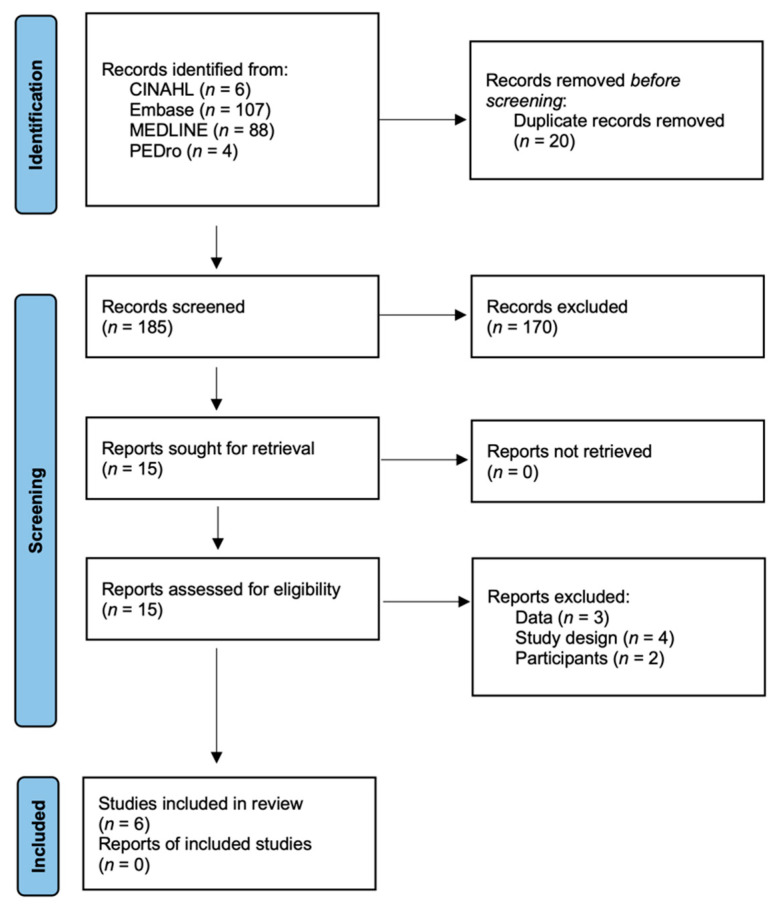
Preferred Reporting Items for Systematic Reviews and Meta-Analysis flow diagram.

Altogether, 279 papers were identified using the four databases (Figure 1). Duplicate studies were classified using Excel, and 20 studies were excluded. Altogether, 170 studies were excluded for not conforming to the eligibility criteria. Following the review of full texts, three studies with inadequate data, four with inappropriate study designs, and two with an inadequate number of participants were excluded. Finally, six studies were selected in this systematic review and meta-analysis [24,25,26,27,28,29].

### 3.2. Assessment of Methodological Quality

**Figure 2:**
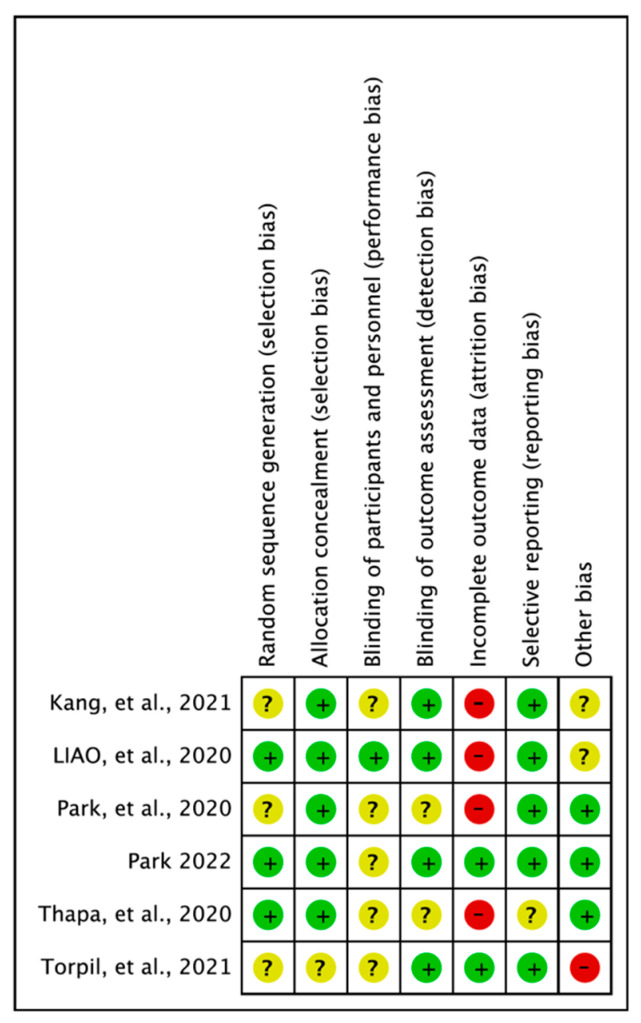
Risk of bias summary: review of authors’ judgments about each item for each included study. Kang et al., 2021 [24], Liao et al., 2020 [25], Park 2022 [26], Park et al., 2020 [27], Thapa et al., 2020 [28], Torpil et al., 2021 [29].

The results of quality assessment were as follows: random sequence generation (low: 3, uncertain: 3), allocation concealment (low: 5, uncertain: 1), blinding of participants and personnel (low: 1, uncertain: 5), blinding of outcome assessment (low: 1, uncertain: 5), incomplete outcome data (low: 2, high: 4), selective reporting (low: 5, uncertain: 1), and other biases (low: 3, uncertain: 2, high: 1). For other biases, items such as lack of sample size calculations, differences in baseline characteristics, and lack of study protocol registration were assessed as uncertain or high [30] (Figure 2).

### 3.3. Virtual Reality for Individuals with Mild Cognitive Impairment

The six RCTs from this systematic review included 279 individuals with MCI. The interventions included VR without distinguishing between immersive and semi-immersive types. The treatment duration varied from 4 weeks to 3 months (Table 1). Cognitive function was classified into global cognition (Mini-Mental State Examination, Montreal Cognitive Assessment [31], and Loewenstein Occupational Therapy Cognitive Assessment-Geriatric [32]), working memory (Trail Making Test-part A [33,34] and digit span test [35]), executive function (Trail Making Test-part B [33,34], Digit Symbol Substitution Test, Weschsler Adult Intelligence Scale-revised Block Design Test [36], and Executive Interview 25 [37]), memory function (Seoul Verbal Learning Test [38] and California Verbal Learning Test [39]), and attention (Stroop test [40]) for outcome measurement (Table 1 and Table 2).

### 3.4. Effectiveness of Virtual Reality in Treating Mild Cognitive Impairment

**Figure 3:**
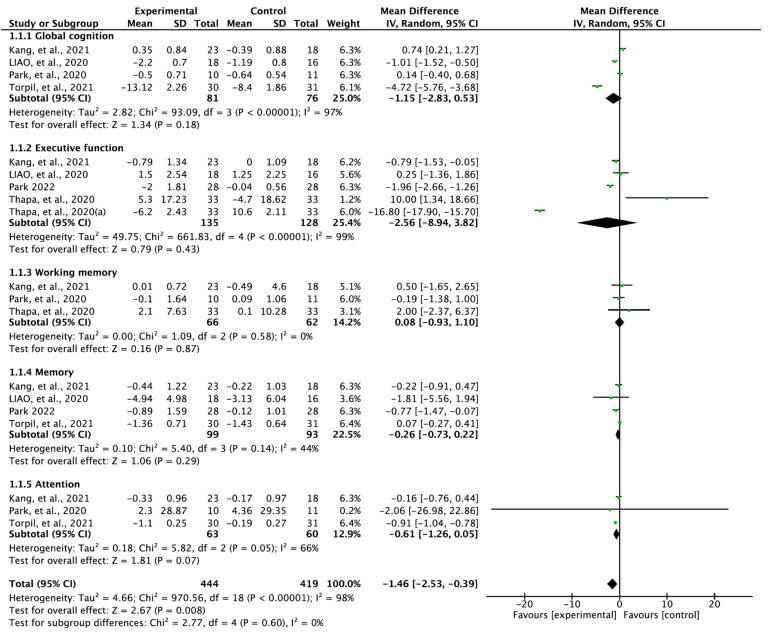
Forest plot studying the effect of virtual reality on cognitive function. Thapa, et al. 2020 (a) Digit Symbol Substitution Test. Kang et al., 2021 [24], Liao et al., 2020 [25], Park 2022 [26], Park et al., 2020 [27], Thapa et al., 2020 [28], Torpil et al., 2021 [29].

The studies showed a significant positive effect of therapeutically applied VR on the cognitive function of individuals with MCI (MD = −1.46; 95% confidence interval (CI): −2.53 to −0.39; heterogeneity: χ^2^ = 970.56, df = 18, I^2^ = 98%; overall effect: Z = 2.67, *p* = 0.008). Subcategories such as cognitive function (global cognition, working memory, executive function, memory function, and attention) were analyzed using subgroup analysis (Figure 3). There was no significant improvement in global cognition (MD = −1.15; 95% CI: −2.83 to 0.53), executive function (MD = −2.56; 95% CI: −8.94 to 3.82), working memory (MD = 0.08, 95% CI: −0.93 to 1.10), memory function (MD = −0.26, 95% CI: −0.73, 0.22), and attention (MD = −0.61, 95% CI: −1.26 to 0.05) when compared with the control group.

### 3.5. Publication Bias

In this review, six studies were synthesized for meta-analysis according to eligibility criteria. The Cochrane Review [41] recommended that publication bias is not appropriate when fewer than 10 studies are synthesized, and thus it was not analyzed.

## 4. Discussion

In the present review, we performed qualitative and quantitative analyses by synthesizing RCTs that involved therapeutic application of VR in individuals with MCI. To the best of our knowledge, this is the first meta-analysis to classify the cognitive function and analyze the improvements in each subcategory.

Therapeutic use of VR had positive effects on cognitive function in individuals with MCI (MD = −1.46, 95% CI: −2.53 to −0.39; overall effect: Z = 2.67, *p* = 0.008). However, there was no significant improvement in the subcategories such as global cognition (MD = −1.15, 95% CI: −2.83 to 0.53), executive function (MD = −2.56, 95% CI: −8.94 to 3.82), working memory (MD = 0.08, 95% CI: −0.93 to 1.10), memory function (MD = −0.26, 95% CI: −0.73 to 0.22), and attention (MD = −0.61, 95% CI: −1.26 to 0.05) when compared with the control group. Our results differed from those reported in previous meta-analyses [14,15,16,17,18,19,20,21], which showed significant improvements in global cognition. A previous meta-analysis showed significant improvements in executive function [19,20,21] and memory function [15,19]. However, another meta-analysis reported no positive effects on memory function [17,20,21], execution function [17], and attention [17,21].

Some systematic reviews have reported results similar to those in the present review. However, the overall results in the present review were not consistent with those from previous reviews. This discrepancy might have been due to differences in methodological factors (determining the effect of VR alone through RCTs, the difference in search strategy, and lack of distinction between immersive and semi-immersive VR) and analyses (cognitive function was subdivided into categories, and each assessment tool was analyzed according to this classification). However, this does not imply that the results of the present review are absolute. The present review did not differentiate between immersive and semi-immersive VR images. According to a systematic review by Yu, Li and Lai [15], the semi-immersive and non-immersive types are more effective than the immersive type, since immersive technologies can be complex and difficult for individuals with MCI [42].

Although there was no significant improvement in the treatment effect of VR when compared with the control group, application of VR in the treatment environment might have a large potential impact in the future. VR elicits virtual sensations through the simulation of a virtual body [43], which can be provided with an immediate response to reduce compensatory movements by enhancing movement control as a feedback system [44]. Therefore, the provision of feedback should improve cognition and daily life functions by stimulating cognitive and motor domains [45]. Moreover, from a neuroscientific perspective, sharing the basic mechanism of the brain in VR should elicit physiological and psychological responses [46]. This involves observing the movement of the body in a virtual environment, which induces changes in muscle activity, heart rate, and stress [46].

Although the efficacy of VR-based cognitive training might decrease with age [47], it is suggested to be more effective when combined with physical training [48], since physical training increases brain-derived neurotrophic factor, which is concentrated in the hippocampus [49,50]. It has also led to activation of the frontal lobe in studies using magnetic resonance imaging [51]. Moreover, we found that combining VR-based training with physical training could be more effective [52] and could improve neuroplasticity in the ventral striatum by linking the motor and cognitive circuits [53]. Finally, from a functional point of view, the ability to switch between different tasks and to focus on tasks in a VR program that requires visual ability, attention [20], and real-time feedback stimulation should have a positive effect on individuals with MCI [48].

In the present systematic review and meta-analysis, therapeutic application of VR in individuals with MCI was more effective in improving cognitive function when compared with the control group. Despite the contradictory results, none of the subcategories of cognitive function showed significant improvement. However, the potential impact of immersive technology on enhancing the feedback systems and the neuroscientific mechanisms that can act as beneficial stimuli have identified therapeutic application of VR as an area that requires further study. This review has several limitations. Generalizability of a comprehensive review involving only six studies might be limited. We did not consider the different types of VR in the analysis. The intensity of interventions (duration and training protocol) was inconsistent in the present review. Finally, there was a significant improvement in cognitive function, but it was associated with a very high heterogeneity.

## 5. Conclusions

Therapeutic application of VR in individuals with MCI contributes to the improvement of cognitive function. However, its efficacy in some of the subcategories of cognitive function (global cognition, working memory, executive function, memory function, attention) is unclear. Further studies will require customized programs based on individual subcategories of cognitive function.

## Figures and Tables

**Table 1:** Classification of cognitive function.

Cognition	Kang et al., 2021 [24]	Liao et al., 2020 [25]	Park 2022 [26]	Park et al., 2020 [27]	Thapa et al., 2020 [28]	Torpil et al., 2021 [29]
Global cognition	MMSE	MoCA		K-MMSE		LOTCA-G
Working memory	TMT-A			Digit span	TMT-A	
Executive function	TMT-B	EXIT-25	WAIS-BDT		DSST, TMT-B	
Memory function	SVLT	CVVLT	SVLT			LOTCA-G
Attention	Stroop test			Stroop test		LOTCA-G

CVVLT, Chinese version of the California Verbal Learning Test; DSST, Digit Symbol Substitution Test; EXIT-25, Executive Interview 25; K-MMSE, Korean version of the Mini-Mental State Examination; LOTCA-G, Loewenstein Occupational Therapy Cognitive Assessment-Geriatric; MMSE, Mini-Mental State Examination; MoCA, Montreal Cognitive Assessment; SVLT, Seoul Verbal Learning Test; TMT-A, Trail Making Test-Part A; TMT-B, Trail Making Test-Part B; WAIS-BDT, Weschsler Adult Intelligence Scale-Revised Block Design Test.

**Table 2:** Characteristics of the included trials.

Study	Sample Size	Duration	Intervention	Authors’ Conclusion
Kang et al., 2021 [24]	EG = 23 CG = 18	4 weeks	EG = VR cognitive training twice a week, total eight sessions, fully immersive 3D setting CG = usual care	Fully immersive VR cognitive training had positive effects on the visuospatial function, apathy, affect, and quality of life, and increased frontal-occipital functional connectivity in older individuals in a predementia state.
Liao et al., 2020 [25]	EG = 18 CG = 16	12 weeks	60 min per session, three sessions per week, total of 36 sessions EG = VR-based PCT; take mass rapid transit, look for a store, kitchen chef, convenience-store clerk CG = PCT	VR-based physical and cognitive training improved cognitive function.
Park, 2022 [26]	EG = 28 CG = 28	8 weeks	EG = VR-based spatial cognitive training; 24 sessions (45 min per session, 3 days per week), program in Unity game engine CG = no intervention	VR-based spatial cognitive training might be clinically beneficial for improving spatial cognition and episodic memory in elderly individuals with MCI.
Park et al., 2020 [27]	EG = 10 CG = 11	3 months	EG = Culture-based VR training; 24 sessions (30 min per day, 2 days per week), training with games (Crows and Seagulls, Janggu, Automated Teller Machine, Shopping in the Mart, Fireworks Party, Fruit Cocktail) CG = no intervention	Culture-based VR training programs did not improve cognitive function.
Thapa et al., 2020 [28]	EG = 33 CG = 33	8 weeks	EG = VR; 100 min (three 20 min VR training sessions and three 10 min eye massage and stretching sessions), sessions held three times a week, VR training games (juice making, crow shooting, find the number of fireworks, memory object at the house) CG = HCE; 30–50 min per session, one session per week, total eight sessions	VR-based training improved cognitive and physical function in patients with MCI when compared with controls.
Torpil et al., 2021 [29]	EG = 30 CG = 31	10–12 weeks	45 min per session, two sessions per week, total 24 sessions EG = Cognitive rehabilitation plus VR; Microsoft Kinect for PC without immersion (Boxing Trainer, Jet Run, Superkick, Air Challenge) CG = cognitive rehabilitation	Using VR applications in CR is recommended to improve cognitive function of older adults with MCI.

CG, control group; CR, cognitive rehabilitation; EG, experimental group; HCE, home care education; MCI, mild cognitive impairment; PCT, physical and cognitive training; VR, virtual reality; 3D, three-dimensional.

## Data Availability

Not applicable.
